# Age-specific mortality patterns in Central Mozambique during and after the end of the Civil War

**DOI:** 10.1186/1752-1505-5-8

**Published:** 2011-05-26

**Authors:** Bruce H Noden, R John C Pearson, Aurelio Gomes

**Affiliations:** 1 Department of Biomedical Science, School of Health and Applied Science, Polytechnic of Namibia, Windhoek, Namibia; 2 Department of Community Medicine, West Virginia University, West Virginia, USA (Emeritus Professor; 3 World Learning, Washington, DC, USA

## Abstract

**Background:**

In recent years, vigorous debate has developed concerning how conflicts contribute to the spread of infectious diseases, and in particular, the role of post-conflict situations in the epidemiology of HIV/AIDS. This study details the age-specific mortality patterns among the population in the central provincial capital of Beira, Mozambique, during and after the Mozambican civil war which ended in 1992.

**Methods:**

Data was collected from the death register at Beira's Central Hospital between 1985 and 2003 and descriptively analyzed.

**Results:**

The data show two distinct periods: before and after the peace agreements in 1992. Before 1992 (during the civil war), the main impact of mortality was on children below 5 years of age, including still births, accounting for 58% of all deaths. After the war ended in 1992, the pattern shifted dramatically and rapidly to the 15-49 year old age group which accounted for 49% of all deaths by 2003.

**Conclusions:**

As under-5 mortality rates were decreasing at the end of the conflict, rates for 24-49 year old adults began to dramatically increase due to AIDS. This study demonstrates that strategies can be implemented during conflicts to decrease mortality rates in one vulnerable population but post-conflict dynamics can bring together other factors which contribute to the rapid spread of other infectious diseases in other vulnerable populations.

## Background

In recent years, vigorous debate has developed concerning how conflicts contribute to the spread of infectious diseases, and in particular, the role of post-conflict situations in the epidemiology of HIV/AIDS [1-3]. It has been widely assumed that the disarray accompanying conflicts contributes significantly to the spread of HIV in local population [4]. However, others have shown that the spread of HIV occurs primarily in the post-conflict period when the isolation of the population is removed and the freedom to travel improves [1,4,5]. Central Mozambique is a unique place in which to evaluate the relationship between conflict and mortality related to infectious diseases as an extended civil war ended in 1992. The aim of this retrospective study was to detail the age-specific mortality patterns among the population in the central provincial capital of Beira, Mozambique, during and after the Mozambican civil war.

## Methods

### Setting

Beira, Mozambique, the provincial capital of the central province of Sofala, is situated on the Indian Ocean. Since the 1950s, Beira has served as the major port city for the landlocked neighbouring countries of Zimbabwe, Zambia and Malawi. During the civil war which began in 1976, Beira was relatively isolated, except for the influx of truckers and military personnel from neighbouring countries of Zimbabwe, Zambia and Malawi assisting to keep the 'Beira Corridor' open to trade. When the peace accords were signed in 1992, Mozambique was one of the poorest countries in the world [6]. The city now has a population of 432,000 [7] and is now considered the third largest city in Mozambique.

The Central Hospital of Beira is one of only 3 central hospitals in Mozambique [8]. While serving as a primary (local) and secondary (provincial) medical centre for one of the largest urban populations in Mozambique, it also acts as a tertiary referral centre for Central Mozambique [9]. It is the main training centre [8] with an associated medical school (Catholic University of Mozambique Faculty of Health Sciences) since 2001. Therefore, it is a major catchment hospital for critical cases from which mortality studies are appropriate.

### Data collection

Data for this study was collected from the death register at Beira's Central Hospital. All deaths are required by law to be registered before burial. Therefore, the data from the Central Hospital ensures that most deaths occurring between 1985 and 2003 were recorded.

The population of Beira was not precisely known until the first census in 1997. At that time, general estimates were made for both the province of Sofala (of which Beira is the capital) and the city of Beira [10]. The percentage distribution of mortality was accordingly compiled using published age distribution categories [11].

This study was part of an outreach program of the Faculty of Health Sciences of the Catholic University of Mozambique. The medical students involved in the data collection were closely supervised by experienced nurses and the complete anonymity of the names in the register was ensured at all times. The overall supervision was done by an experienced physician.

For this study, data from the death registers was transferred to worksheets providing information of register number, sex, age, place of birth, place of residence, date of death and diagnosis. All data was then coded using ICD-10, three digits, entered into Excel worksheets and converted to an Access database, closely supervised by a physician. For the current study, only age was analyzed. Where the age at death was not known, the diagnosis was checked, and if indicative of the age category (e.g. born dead or premature), the death was recorded. If not, 'unknown' age was recorded. Analysis was performed using EPI Info version 3.2 and graphs created using Excel 2007.

## Results

**Figure 1:**
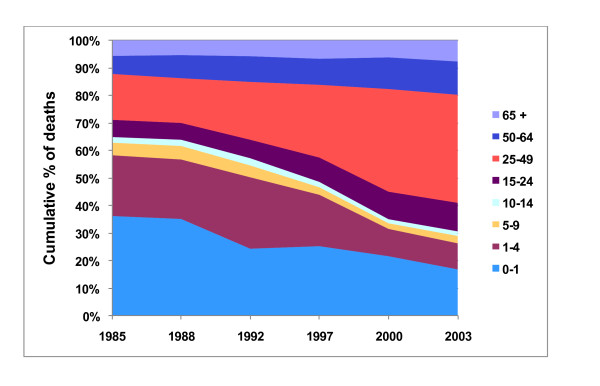
**Age-specific mortality in the city of Beira, Mozambique, 1985-2003**.

**Figure 2:**
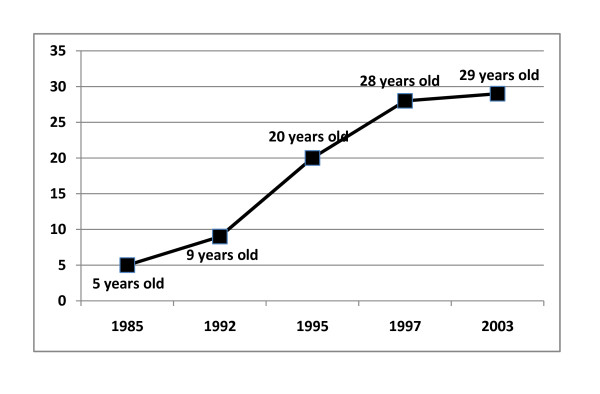
**Median age of mortality in the city of Beira, Mozambique, 1985-2003**.

**Figure 3:**
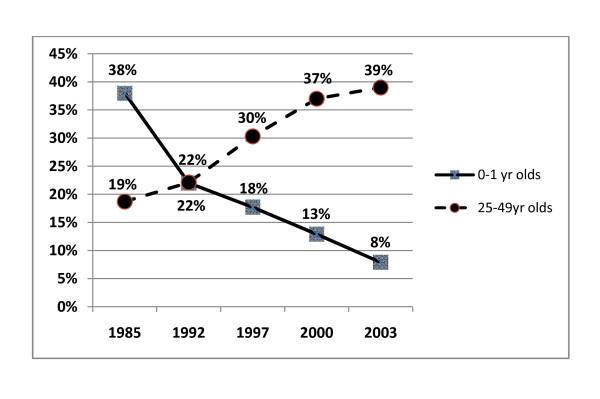
**Mortality rates (%) of prenatal (ages 0-1) and adults (ages 25-49) in Beira, Mozambique, 1985-2003**.

Between 1985 and 2003, there was a dramatic shift in the age-specific mortality patterns in populations in Beira, Mozambique (Figure 1). This shift was most obviously observed in the increase in the median age of mortality (Figure 2). As infant mortality rates were decreasing, there was a substantial increase in mortality among 25-49 year olds (Figure 3). In terms of actual population, total numbers of deaths in Beira increased 2.3 × between 1985 (2450) and 2003 (5605). While Under-5 mortality remained relatively constant (n = 1429 (1985) to n = 1479 (2003), mortality among the 24-49 year olds went from 408 (1985) to 2197 (2003) (an increase of 5.4×). By 2003, mortality rates among the 15-49 year olds accounted for ~60% of all deaths (Figure 1).

**Figure 4:**
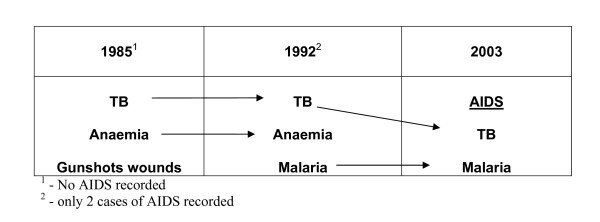
**Top 3 causes of mortality among 15-49 year olds in Beira, Mozambique, 1985, 1992 and 2003**.

In 1985, main causes of death among 15-49 year olds were tuberculosis (15%), anaemia (8%), and gunshots wounds (7%) (Figure 4). Patterns changed slightly in 1992 with tuberculosis (14%), anaemia (8%), malaria (8%), and gastroenteritis (not cholera) (6%). Only two deaths were ascribed to HIV/AIDS. By 2003, AIDS/Immunodeficiency accounted for 29% of deaths followed by tuberculosis (18%) and malaria (18%).

The infant mortality rate in Beira was 65.8 (per 1000) in 1997, but fell to 38.2 in 2001 and 38.0 in 2003. Also, the child mortality rate (1-4 year olds) fell from 13.0 (2001) to 9.4 (2003). While the mortality rates for 5-14 year olds remained stable (1.7/1000) between 2001 and 2003, there was a small rise in the mortality rates among 15-24 year olds from 3.6 to 5.0 and even larger increases for the 25-49 year old group (9.0 to 13.1), the 50-64 age group (11.2 to 23.6) and in those older than 65 (40.3 to 57.8).

Interestingly, while the Beira infant mortality rate (per 1000) fell from 65.8 in 1997 to 38.2 in 2001, the overall mortality rate for Sofala province dropped from 144 (1997) to 128 (2003). When Beira was excluded, the mortality rate for the rest of province of Sofala hardly changed from 167 (1997) to 164 (2003).

## Discussion

The data suggest two major trends occurred in the Mozambique between 1985 and 2003. These trends demonstrate the issues impacting conflict and post-conflict situations [1,4,5]. First, during the civil war (before 1992), mortality was highest among children below 5 years of age (including still births), accounting for two thirds of all deaths. This reduction is confirmed by Cutts et al. [12] in an epidemiological study done in the communities of Beira during that period. The low mortality rate among 15-49 year olds during the same period is notable, particularly during a time of conflict. Because of the poor state of the health services in Central Mozambique due to the destruction of most rural health posts, the primary focus of humanitarian organizations (i.e. International Committee of the Red Cross (ICRC)) was to reduce maternal and child-related mortality [13]. The overall effect was a dramatic decrease in mortality in the under-5 children in Central Mozambique [13] and Southern Mozambique [14] which is also observable in this Beira Central Hospital data set.

The post-conflict trend occurred 5 years after the civil war ended (post-1992) when the mortality pattern dramatically shifted to those older than 15 years old, with the greatest impact occurring among 25-49 year olds. During this period, the main cause of death was attributed to AIDS. Until the late 1996, the epidemiology of HIV infections in Central Mozambique was unknown. Routine testing for HIV among pregnant females was implemented in Maputo (the capital of Mozambique) in 1986. However, it did not begin in Beira until 1996 [15]. During these 10 years of non-monitoring, HIV expanded dramatically in Central Mozambique. By 1996, 17% of the pregnant women in Beira were already infected with HIV. This was also found in other major urban centers in Central Mozambique, Chimoio and Tete, with HIV prevalences of 19.2% and 22.5%, respectively among pregnant women. As of 2006, the HIV prevalence rate in Mozambique was 16.2% [15] with the highest HIV prevalence registered in the central province of Sofala with an official rate of 26.5%, with Beira registering 38% [15].

From this data, it is apparent that during the conflict stage, the government and humanitarian groups focused to ensure appropriate health care to the under-5 cohort in the early 1990s (a component more easily addressed during the conflict stage). During the same conflict period, unmonitored HIV spread rapidly among vulnerable populations in Central Mozambique. While truck drivers and military personnel from neighbouring countries with high HIV prevalence [16-19] may have brought the HIV to relatively isolated urban populations [1], local populations were primed for an HIV epidemic because of high prevalence of untreated STIs [20,21]. Additionally, after the peace accords in 1992, large numbers of refugees returned from neighbouring countries [16-18] with unknown HIV prevalence. We believe that the mixing of HIV-infected refugees into the these relatively isolated communities with high prevalence of untreated STIs [4,22] contributed to the explosion of HIV which culminated in increased AIDS mortality in Central Mozambique in mid-to-late 1990s. Finally, the influx of UN peacekeepers in 1993 with their perceived wealth and the creation of a large commercial sex industry in Beira, may have also contributed to the spread of HIV in the community [4]. The Mozambique national program for HIV prevention only began to respond in Central Mozambique in 1999 while it had been actively working in Maputo since 1986.

While possible limitations occurred, every effort was made to reduce their effects. One limitation is the possible bias introduced into the analysis by misreporting of the ages at death, particularly that of rounding of ages at death. While common for mortality-focused studies [23-25], this can underestimate the mortality of some age groups and overestimate for others. However, because of the large sample size used and the completeness of the data set, we believe the trends were not altered significantly by this possible limitation.

Another limitation involves the completeness of the death registry from only one institution, even one as regionally significant as Beira Central Hospital. A law requires the registration of all deaths occurring in the Beira area at the mortuary of the Central Hospital in order to receive a certificate for a burial plot assigned by an area municipal office [9]. It is not possible, however, to control for those who die at home and are buried in 'informal' cemeteries. In their 1996-1997 study in Beira, Songane & Bergstrom [9] reported a high rate of underreporting of maternal deaths in the official register. Apart from the four public cemeteries (one which is already full), there are 10^+ ^private cemeteries in which mortality information is not documented. The fact that there were 2.3 × more recorded deaths in 2003 compared with 1985 leads one to consider that misreporting could have affected some of the patterns observed in our study, particularly early during the conflict phase.

Another factor involves the contribution of reduced under-5 mortality on the increased proportion of mortality observed among 24-49 year olds between 1992 and 2003. From the data, we do not believe that this reduction had much of an effect. Over the period of study, our data records that under-5 mortality numbers stayed relatively constant (while the proportion in the mortality population decreased) whereas the total mortality among 15-49 year olds increased 5.4×. Where the effect of a decrease under-5 mortality (observed locally [13] and nationally [26]) may be seen is in the slight increase of life expectancy rates in Mozambique from 42.7 (1985) to 47.6 (2003) [27].

It is notable that during the war period in the Beira Central Hospital records, 58% (1985) to 50% (1992) of the mortalities registered were children under 5 years old. While gender was not analyzed for this study, maternal mortality in Beira was estimated at 10.3% by Cutts et al. [12] but the authors felt their informal methods underestimated the true number. Even with their conservative estimate, the combined child and maternal mortality in Beira during the civil war years was over 60% of the total mortality.

During the late 1980s, Central Mozambique was involved in civil war which systematically focused on rural health services [28]. Rural clinics were systematically destroyed and health workers were kidnapped or killed. Ambulances and health vehicles were routinely attacked and supply support systems to rural clinics were seriously affected. Between 1983 and 1987, the mortality rates of displaced persons were 2.7-6.3 times higher than the national average. By 1987, trauma was the leading cause of death among adults in Tete province. This is observable in our data set with the third highest cause of mortality in Beira in 1985 being gunshot wounds. During this conflict period, 1.2 million persons were displaced from their homes [28]. Because of this massive disruption, Beira filled with persons escaping from the unstable situation with many being women and children. In 1993, Cutts et al. [12] determined that two major risk factors for child mortality in Beira were recent migration (child born outside Beira) and absence of a father. This indicates that women may have been searching for sanctuary in Beira while men were either fighting or working in neighboring countries. So, agreeing with conclusions raised by O'Hare and Southall [29], this data re-emphasizes the impact of conflict on maternal and child health and leads to a special appeal to wealthy countries who sell the weapons to the warring groups in developing countries to consider afresh what it means to 'do no harm' at the expense of getting income from weapon sales. With the end of the war, there may have been a dramatic shift in population with men returning which could have helped explain some of the patterns observed in the late 1990s-early 2000s.

Even including the other causes among the 24-49 year olds in Beira, HIV/AIDS was the main cause of death in the post-conflict period. The epidemic began 'silently' in the population in the mid-1980s. By 2000, the HIV seroprevalence among pregnant women in Beira was just under 30% [30]. Until the mid-2000s, it was considered shameful to put AIDS as the cause of death on the register. It is interesting to note in our data that until the early 2000s, tuberculosis was the main cause of death among 15-49 year olds. It begs the question of how many of those deaths from tuberculosis were actually caused by undiagnosed HIV/AIDS. However, while both tuberculosis and malaria were significant among 15-49 year olds in 2003, HIV/AIDS quickly became the leading cause. These mortality patterns among 15-49 year olds were also corroborated by others inside Mozambique [26,31] and in Southern Africa [14].

## Conclusions

In conclusion, while much effort went into reducing the mortality of children under 5 at the close of the Mozambique civil war, a silent HIV epidemic had already entered into relatively isolated communities. Without an HIV prevention strategy in place, the influx of refugees from neighbouring countries with high HIV rates together with local populations with high STI rates worked together to significantly impact adult mortality in the post-conflict state. This demonstrates that during conflicts, resources can be focused to reduce mortality rates in one population cohort. However, the dynamics in the immediate post-conflict period can bring together other factors which contribute to the rapid spread of different infectious diseases in vulnerable populations, thus nullifying the previous gains.

## Competing interests

The authors declare that they have no competing interests.

## Authors' contributions

The three authors participated in designing the study. AG organized the data collection. RJCP carried out the data analysis. BHN drafted the first version of the paper. All authors extensively reviewed all drafts, made comprehensive changes, and approved the final draft.
